# CpG Oligodeoxinucleotides and Flagellin Modulate the Immune Response to Antigens Targeted to CD8α^+^ and CD8α^−^ Conventional Dendritic Cell Subsets

**DOI:** 10.3389/fimmu.2017.01727

**Published:** 2017-12-04

**Authors:** Renan Antonialli, Fernando Bandeira Sulczewski, Kelly Nazaré da Silva Amorim, Bianca da Silva Almeida, Natália Soares Ferreira, Márcio Massao Yamamoto, Irene Silva Soares, Luís Carlos de Souza Ferreira, Daniela Santoro Rosa, Silvia Beatriz Boscardin

**Affiliations:** ^1^Department of Parasitology, Institute of Biomedical Sciences, University of São Paulo, São Paulo, Brazil; ^2^Department of Clinical and Toxicological Analysis, School of Pharmaceutical Sciences, University of São Paulo, São Paulo, Brazil; ^3^Department of Microbiology, Institute of Biomedical Sciences, University of São Paulo, São Paulo, Brazil; ^4^Department of Microbiology, Immunology and Parasitology, Federal University of São Paulo, São Paulo, Brazil; ^5^Institute for Investigation in Immunology (iii), INCT, São Paulo, Brazil

**Keywords:** dendritic cells, hybrid monoclonal antibodies, CpG oligodeoxinucleotides 1826, flagellin, antigen targeting

## Abstract

Dendritic cells (DCs) are antigen-presenting cells essential for the induction of adaptive immune responses. Their unprecedented ability to present antigens to T cells has made them excellent targets for vaccine development. In the last years, a new technology based on antigen delivery directly to different DC subsets through the use of hybrid monoclonal antibodies (mAbs) to DC surface receptors fused to antigens of interest opened new perspectives for the induction of robust immune responses. Normally, the hybrid mAbs are administered with adjuvants that induce DC maturation. In this work, we targeted an antigen to the CD8α^+^ or the CD8α^−^ DC subsets in the presence of CpG oligodeoxinucleotides (ODN) or bacterial flagellin, using hybrid αDEC205 or αDCIR2 mAbs, respectively. We also accessed the role of toll-like receptors (TLRs) 5 and 9 signaling in the induction of specific humoral and cellular immune responses. Wild-type and TLR5 or TLR9 knockout mice were immunized with two doses of the hybrid αDEC205 or αDCIR2 mAbs, as well as with an isotype control, together with CpG ODN 1826 or flagellin. A chimeric antigen containing the *Plasmodium vivax* 19 kDa portion of the merozoite surface protein (MSP1_19_) linked to the Pan-allelic DR epitope was fused to each mAb. Specific CD4^+^ T cell proliferation, cytokine, and antibody production were analyzed. We found that CpG ODN 1826 or flagellin were able to induce CD4^+^ T cell proliferation, CD4^+^ T cells producing pro-inflammatory cytokines, and specific antibodies when the antigen was targeted to the CD8α^+^ DC subset. On the other hand, antigen targeting to CD8α^−^ DC subset promoted specific antibody responses and proliferation, but no detectable pro-inflammatory CD4^+^ T cell responses. Also, specific antibody responses after antigen targeting to CD8α^+^ or CD8α^−^ DCs were reduced in the absence of TLR9 or TLR5 signaling, while CD4^+^ T cell proliferation was mainly affected after antigen targeting to CD8α^+^ DCs and in the absence of TLR9 signaling. These results extend our understanding of the modulation of specific immune responses induced by antigen targeting to DCs in the presence of different adjuvants. Such knowledge may be useful for the optimization of DC-based vaccines.

## Introduction

Dendritic cells (DCs) are innate immune cells specialized in antigen presentation to naïve T lymphocytes (1). DCs express pattern recognition receptors (PRRs), such as toll-like (TLRs) and nod-like (NLR) receptors, which recognize pathogen- or damage-associated molecular patterns (PAMPs or DAMPs), respectively (2). After pathogen contact, DCs mature, produce cytokines, and upregulate costimulatory molecules that prime CD4^+^ and CD8^+^ T cell responses, and stimulate B cells to produce antibodies (3–5). Thus, DCs play a central role in immunity, promoting, and controlling the adaptive immune response during inflammation (6).

Dendritic cells are a heterogeneous lineage of cells that differentiate from bone-marrow precursors and migrate to different regions of the body, such as blood, thymus, liver, lymphoid organs, spleen, and skin (7–9). DCs can be divided in two main subtypes: plasmacytoid DCs that are mainly associated with antiviral response and conventional DCs mainly related with antigen presentation (7). Classically, murine conventional spleen DCs (CD11c^+^MHCII^+^) can be classified according to the expression of the CD8 molecule alpha chain. CD8α^+^ DCs (CD11c^+^CD8α^+^) are mainly associated with cross-presentation to CD8^+^ T cells, while CD8α^−^ DCs (CD11c^+^CD8α^−^) with antigen presentation to CD4^+^ T cells (10–12). More recently, conventional DCs were classified into two distinct subtypes based on their ontogeny: the conventional type 1 DCs (cDC1s, CD11c^+^CD26^+^XCR1^hi^CD172a^lo^IRF8^hi^IRF4^lo^) and conventional type 2 DCs (cDC2s, CD11c^+^CD26^+^XCR1^lo^CD172a^hi^ IRF8^lo^IRF4^hi^) (13). Evidences support the notion that the CD8α^+^ DCs correspond to cDC1s, while CD8α^−^ DCs correspond to cDC2s (14, 15).

In addition to the markers mentioned above, conventional DCs also express endocytic receptors that belong to the C-type lectin family. While the CD8α^+^ DCs express the DEC205 receptor (16), the CD8α^−^ DCs express a receptor known as DCIR2 (17). αDEC205 and αDCIR2 monoclonal antibodies (mAbs) have been successfully used to target antigens to CD8α^+^ DCs and CD8α^−^ DCs, respectively (18–20). This is accomplished by fusing the antigen of interest to the carboxyl terminus portion of the αDEC205 or αDCIR2 heavy chains. The result is a hybrid mAb that, once administered to mice, delivers the antigen of interest to the DCs *in vivo* and consequently promotes antigen processing and presentation (21). Nevertheless, the use of this strategy to induce an immune response against proteins expressed by different pathogens requires the administration of an adjuvant to mature the DCs, and avoid the development of tolerance (22, 23).

The αCD40 agonistic mAb was frequently used as an adjuvant in immunizations using αDEC205 and αDCIR2 fusion mAbs to promote DC maturation (24) and robust adaptive immune responses (12, 18, 25, 26). Furthermore, PRR ligands have also been used to mature DCs. Polyinosinic:polycytidylic acid (poly (I:C)) is a TLR3 and MDA-5 (melanoma differentiation-associated gene 5) ligand that has been largely used together with hybrid mAbs in protocols intended to target antigens to DCs, especially through the DEC205 receptor (19, 20, 26–28). In fact, it was shown that poly (I:C) administered together with an αDEC205 fusion mAb was the best adjuvant to induce potent IFN-γ-producing CD4^+^ T cells (27, 29).

Despite the use of αCD40 agonistic mAb and poly (I:C) as adjuvants, the search for new adjuvants that may be used together with the hybrid mAbs is still relevant, especially when targeting the CD8α^−^ DCs with the αDCIR2 mAb. Here, we analyzed two other adjuvants in the context of DC targeting. We studied the immune response induced after antigen targeting to CD8α^+^ and CD8α^−^ DCs using CpG oligodeoxynucleotides (CpG ODN) or bacterial flagellin as adjuvants. CpG ODN are PAMPs formed by an unmethylated DNA motif present in microbes that are recognized by TLR9, an intracellular receptor anchored in the endosome internal membrane (30, 31). Flagellin is the main component of bacterial flagellum, and it is recognized by extracellular TLR5 (32, 33) and by the intracellular NLR receptors Naip5 (34) and NLRC4 (35). While both TLRs (5 and 9) signal through the same pathway that involves MyD88 activation followed by NF-κB translocation to the nucleus and subsequent pro-inflammatory cytokine production (36), Naip5 and NLRC4 activate the caspase-1 cascade that culminates in the release of inflammatory cytokines such as IL-1β and IL-18 (34, 35). Due to their potent adjuvant effects, both CpG ODN (37) and flagellin (38, 39) have already been used as adjuvants in a number of clinical trials.

Although CpG ODN and flagellin are well-described adjuvants, their use in DC-targeted vaccination protocols may be further explored. In this paper, we hypothesized that the use of different adjuvants together with antigen targeting to the CD8α^+^ and CD8α^−^ DC subsets might induce differential immune responses based on the DC subtype biology. We used recombinant flagellin as a TLR5 ligand and synthetic CpG ODN as TLR9 ligands. In addition, we investigated the direct role of TLR5 or TLR9 signaling using knockout mice to analyze the influence of their signaling specifically on antigen targeting to CD8α^+^ and CD8α^−^ DCs. Previous studies showed that CD8α^+^ and CD8α^−^ DCs promote CD4^+^ T cell differentiation into diverse Th subsets, indicating that different DC subtypes are diverse in priming naïve T cells suggesting biological differences between them (40–42).

Using a *Plasmodium vivax* protein fused to a well-described CD4^+^ T cell epitope (43), we tested the influence of the adjuvant on cellular and humoral immune responses after antigen targeting to DCs. The antigen is composed by the C-terminal 19 kDa fragment of the Merozoite Surface Protein 1 (MSP1_19_) of *P. vivax* fused to a Pan allelic DR epitope (PADRE) (44, 45) in a construct known as MSP1_19__PADRE. Targeting of MSP1_19__PADRE to different DC subsets allows us to study the humoral immune response through the evaluation of anti-MSP1_19_ antibody titers, as well as, the specific CD4^+^ T cell response using the PADRE epitope.

Our results demonstrate that antigen targeting to CD8α^+^ or CD8α^−^ DCs in the presence of flagellin or CpG ODN induce different immune responses that may be linked to the differential activation of these DC subtypes promoted by TLR5 or TLR9 engagement and signaling. In summary, humoral immune responses were successfully induced after antigen targeting to both DC subsets in the presence of either CpG ODN or flagellin. CpG ODN was more suitable to induce specific CD4^+^ T cell proliferation and pro-inflammatory cytokines when the antigen was targeted to CD8α^+^ DCs. TLR9 signaling was essential for this response. On the other hand, flagellin induced more pronounced CD4^+^ T cell proliferation when the antigen was targeted to the CD8α^−^ DC subset. TLR5 signaling did not seem to play a major role in this response. The results presented here contribute to shed more light on the use of different adjuvants associated with DC targeted vaccines.

## Materials and Methods

### Mice

C57BL/6 mice of both sexes, and 5- to 9-week-old, were bred at the Isogenic Mouse Facility of the Parasitology Department, University of São Paulo, Brazil. C57BL/6 background TLR5-deficient (KO) (46) and TLR9 KO (47) were kindly provided by Dr. Michel C. Nussenzweig (The Rockefeller University, USA), and bred and used at the same conditions as the C57BL/6 mice. All experimental procedures and animal handling were performed in accordance with the National Institutes of Health Guide for the Care and Use of Laboratory Animals and with the Brazilian National Law on animal care (11.794/2008). The Institutional Animal Care and Use Committee (CEUA) of the University of São Paulo approved all procedures under the protocol number 082.

### Cloning and Expression of the Fusion mAbs and Recombinant Protein Production

The MSP1_19__PADRE sequence was amplified from the pET14b-MSP1_19__PADRE plasmid previously described (43) using forward (5′-GGCTCGAGGAGTTCGGTAGGTTCATGAGCTCCGAGCACACATG-3′) and reverse (5′-GGGCGGCCGCTTATTGCTCAGCGGTGGCAG-3′) primers. Underlined sequences indicate *Xho* I and *Not* I restriction sites, respectively. After amplification using Phusion High-Fidelity DNA Polymerase (New England Biolabs), the insert was digested with *Xho* I and *Not* I, and cloned in frame with the mouse anti-DEC205 (NLDC145 clone), anti-DCIR2 (33D1 clone), or isotype control (GL117 clone) heavy chain carboxyl terminus. The original plasmid constructs are described elsewhere (12, 22). Plasmids pDEC-MSP1_19__PADRE, pDCIR2-MSP1_19__PADRE and pISO-MSP1_19__PADRE were then generated. These plasmids and the plasmids encoding their respective light chains were amplified in DH5α bacteria and subsequently purified in large scale using the QIAGEN Maxi Prep Kit (Qiagen). Transient transfection in human embryonic kidney (HEK) 293T (ATCC No CRL-11268) cells was performed exactly as described elsewhere (19). After purification with protein G beads (GE Healthcare), fusion mAbs were dialyzed in PBS, filtered, and had their concentrations estimated by Bradford assay (Pierce). Samples were stored at −20°C until use.

To analyze the cellular and humoral immune responses after immunization with the fusion mAbs, we produced recombinant MSP1_19_ and MSP1_19__PADRE proteins exactly as described by Cunha et al. (48) and Rosa et al. (43), respectively.

### Fusion mAbs Integrity Evaluation and Binding Assay

The integrity of the purified fusion mAbs was assessed in 12% SDS-PAGE gels under reducing conditions as previously described (28).

The binding assay was performed using Chinese hamster ovary (CHO) cells expressing the mouse DEC205 or DCIR2 receptors. These cells were kindly provided by Dr. Michel C. Nussenzweig (The Rockefeller University, USA). Before use, cells were detached with 1× PBS containing 1 mM of EDTA for 10 min at 37°C. EDTA was neutralized with 500 µL of fetal bovine serum, and cells were washed three times with PBS 1×. One hundred thousand CHO cells expressing each receptor were incubated with 5, 0.5, or 0.05 µg/mL of each fusion mAb on ice for 40 min. Cells were then washed twice with PBS plus 2% fetal bovine serum (Life Technologies) and incubated with anti-mouse IgG-Alexa Fluor 488 (Thermo Scientific) for 40 min on ice. After two additional washes, 20,000 events were acquired using BD LSRFortessa flow cytometer (BD Biosciences).

This assay was also performed on splenocytes isolated from C57BL/6 naïve mice. Five million splenocytes were initially incubated with anti-CD16/32 (BD Fc Block) for 15 min and then incubated with 5, 0.5, or 0.05 µg/mL of each fusion mAb on ice for 40 min. After two washes, biotinylated anti-CD3 (clone 145.2C11), anti-CD49b (clone DX5) and anti-CD19 (clone 1D3) were incubated on ice for 40 min. Splenocytes were then washed twice and incubated with anti-IgG1-PE (clone A85-1), anti-CD11c-BV421 (clone N418), anti-MHCII (I-A/I-E)-FITC (clone 2G9), anti-CD8α-APC (clone 53–67), streptavidin-PerCP, and Live and Dead Aqua (Thermo Fisher Scientific) for 40 min on ice. All antibodies were purchased from BD Biosciences. One million events were acquired using BD LSRFortessa flow cytometer (BD biosciences). Analyses were performed using FlowJo software (version 9.3, Tree Star, San Carlo, CA, USA).

### Flagellin Production and Purification

The *Salmonella* flagellin FliC*d*, originally produced by the *S*. Muenchen patovar, was produced from a recombinant *S*. Dublin strain exactly as described previously (49) and its concentration was determined by the BCA assay (Pierce). Purity was monitored by 12% polyacrylamide gels stained with Coomassie Blue (Amresco). LPS was removed using detoxi-gel columns (Pierce) according to the manufacturer’s protocol. Residual LPS contamination was monitored using the Limulus Amebocyte Lysate assay (Lonza) and shown to be below 3 EU/μg of protein.

### Immunizations

Groups of five animals were immunized with 5 µg of each mAb administered intraperitoneally (i.p.) combined with either 25 μg/animal of CpG ODN 1826 (Invivogen) or 5 μg/animal of *Salmonella* flagellin. Two doses were administered with a 30-day interval between each one. Five days before and 14 days after the administration of the booster dose, sera were collected. The cellular immune response was analyzed 20 days after the administration of the booster dose, when mice were euthanized and had their spleens removed.

### Analysis of MSP1_19_-Specific Antibodies

The presence of anti-MSP1_19_ specific total IgGs, or IgG1, IgG2b, IgG2c, and IgG3 subclasses was detected by ELISA exactly as previously described (28). Antibody titers were normalized in a log10 scale considering the highest serum dilution showing an OD_490_ > 0.1. The IgG1/IgG2c ratio was calculated by dividing the mean values of the highest serum dilution obtained for IgG1 by the mean value of the highest serum dilution obtained for IgG2c without normalization.

### CFSE-Based Proliferation Assay and Detection of Cytokine-Producing Cells by Intracellular Staining

Splenocytes were isolated and processed as previously described (19, 28). For the proliferation assay, fifty million splenocytes obtained from each group of immunized mice were resuspended in 1 mL of PBS previously heated at 37°C containing 1.25 µM CFDA dye (Vybrant CFDA SE—Cell Tracer Kit, Molecular Probes). The cells were then incubated for 10 min at 37°C, centrifuged at 600 × *g* for 5 min, washed three times, and resuspended in 1 mL of R10 [RPMI supplemented with 10% of fetal bovine serum, 2 mM L-glutamine, 10 mM Hepes, 1 mM sodium pyruvate, 1% vol/vol non-essential aminoacid solution, 1% vol/vol vitamin solution, 5 × 10^−5^ M 2-mercaptoetanol (all from Life Technologies), and 20 µg/mL of ciprofloxacin (Isofarma, Brazil)]. In *U*-shaped 96-well plates (Costar), 3 × 10^5^ cells were stimulated with 1 µg/mL of either MSP1_19__PADRE or MSP1_19_ recombinant proteins in each well and incubated for 5 days at 37°C and 5% CO_2_. After this period, the plates were centrifuged, washed, and the triplicates were combined in a single well for labeling with anti-CD4-PerCP (clone RM 4–5) and anti-CD3-APC.Cy7 (clone 145.2C11) for 40 min on ice. Cells were then washed three times with PBS-FBS (PBS plus 2% fetal bovine serum). One hundred thousand events were acquired using FACS Canto II flow cytometer (BD biosciences). The percent of CFSE low cells was calculated after subtraction of the percent of CFSE low cells in the non-pulsed wells.

Detection of cytokine-producing cells by intracellular staining was performed as described elsewhere (28). Briefly, 1 × 10^6^ splenocytes/well were plated in triplicates in *U*-shaped 96-well plates and pulsed with 5 µg/mL of the recombinant MSP1_19__PADRE protein. As negative control, splenocytes were not pulsed. Incubation was performed in R10 medium containing 2 µg/mL of αCD28 agonist antibody. After incubation for an hour at 37°C and 5% CO_2_, Golgi Plug (Brefeldin A, BD Biosciences) was added to each well (0.5 μg/well). Splenocytes were then incubated in the same conditions for 12 additional hours. Plates were centrifuged for 5 min at 1,000 × *g* and washed twice with PBS-FBS. Cells were stained on ice for 45 min with αCD4-PerCP-Cy5.5 mAb (clone RM 4–5). After three washes with PBS-FBS, cells were fixed and permeabilized for 15 min using the Cytofix/Cytoperm kit (BD Biosciences). After three washes with PermWash buffer (BD Biosciences), the intracellular staining was performed on ice for 45 min using the following mAbs: αCD3-APC-Cy7 (clone 145-2C11), αIFNγ-APC (clone XMG1.2), αIL2-PE (clone JES6-5H4), and αTNFα-PE-Cy7 (clone MP6-XT22). Cells were washed three times with PermWash buffer (BD Biosciences) and resuspended in PBS-FBS. One million events were acquired in a FACS Canto II flow cytometer (BD biosciences). The percent of cytokine producing cells was calculated after subtraction of the percent of cytokine producing cells in the non-pulsed wells. All data were analyzed using FlowJo software (version 9.3, Tree Star, San Carlo, CA, USA).

### Expression of Co-stimulatory Molecules on DC Subsets

Mice were immunized i.p. with 25 μg/animal of CpG ODN 1826 (InvivoGen) or with 5 μg/animal of *Salmonella* flagellin (FliC). 6 h after immunization, mice were euthanized and splenocytes were labeled. Fc receptors were blocked with Fc Block (BD Biosciences) and subsequently stained first with anti-CD19-Biotin (clone 1D3), anti-CD3-Biotin (clone 145.2C11), and anti-CD49b-Biotin (clone DX5) for 40 min on ice. After two washes with PBS-2% FBS, cells were then incubated anti-MHCII (I-A/I-E)-Alexa Fluor 700 (clone M5/114.15.2), anti-CD11c-BV421 (clone N418), anti-CD11b-PE.Cy7 (clone M1/70), anti-CD8α-BV786 (clone 52–67), anti-CD80-FITC (clone 16-10A1), anti-CD86-APC (clone GL1), anti-CD40-PE (clone 1C10), Streptavidin APC.Cy7 (all antibodies and the streptavidin were purchased from BD Biosciences) and Live and Dead Aqua (Life Technologies). Flow cytometry was performed using LSRFortessa (BD Biosciences) and results were analyzed in FlowJo software (version 9.3, Tree Star, San Carlo, CA, USA).

### Statistical Analysis

We used Prism 5.0 (GraphPad, CA, USA) for all the analyses. Regular two-way ANOVA and two-way ANOVA for repeated measures were used for multiple comparisons, followed by Bonferroni’s multiple comparison posttest for comparison of specific groups. *p* < 0.05 was considered significant.

## Results

### αDEC205-MSP1_19__PADRE and αDCIR2-MSP1_19__PADRE mAbs Were Successfully Produced and Bound to Their Respective Receptors

**Figure 1:**
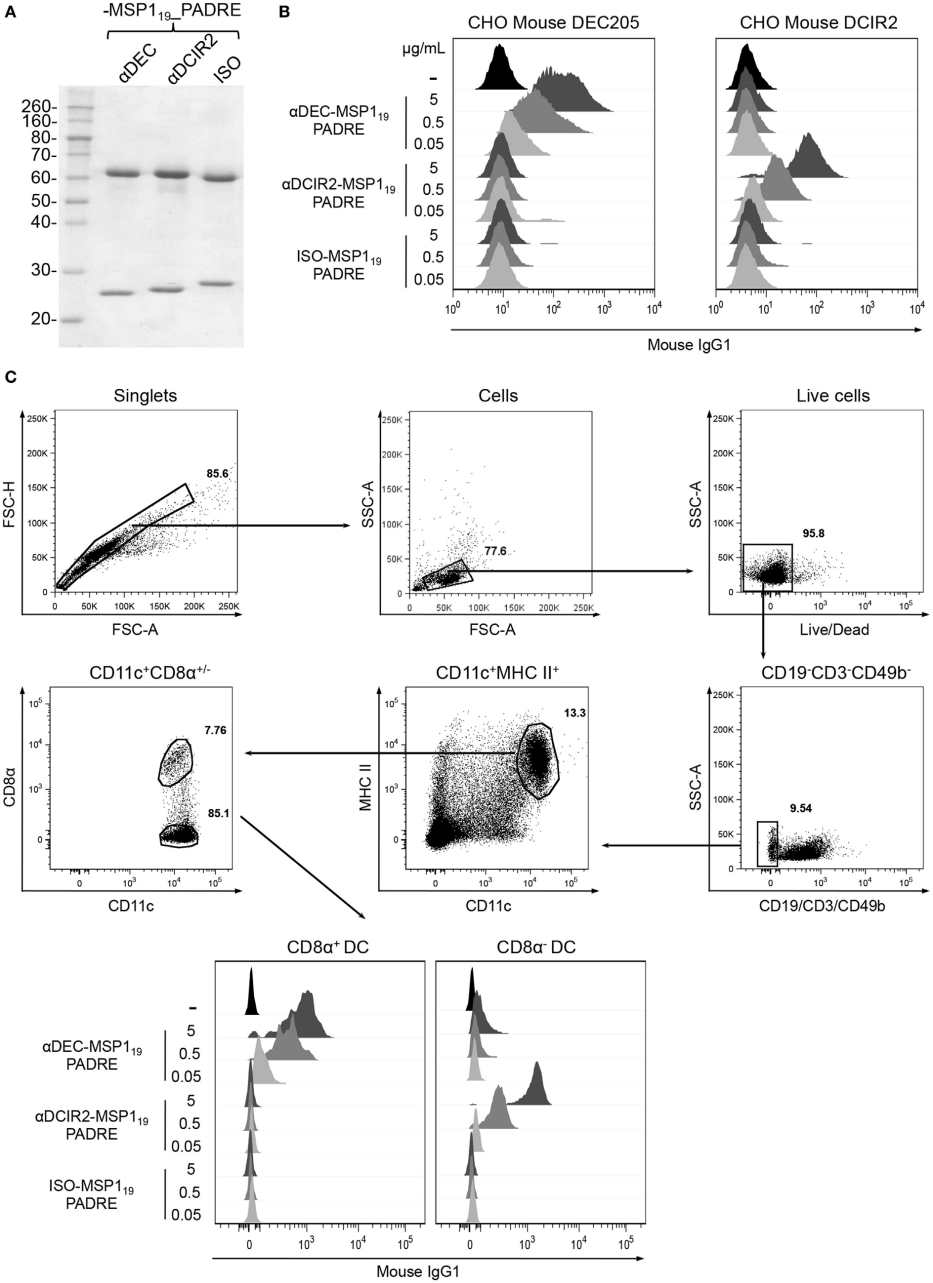
Production and binding of the hybrid monoclonal antibodies (mAbs) αDEC205-MSP1_19__PADRE and αDCIR2-MSP1_19__PADRE to their respective receptors. **(A)** SDS-PAGE under reducing conditions of each hybrid mAb (~1 µg) stained with Coomassie blue dye. Numbers on the left indicate molecular weights in kDa. **(B)** Chinese hamster ovary (CHO) cells expressing the murine DEC205 (left) or DCIR2 (right) receptors were incubated with 0.05, 0.5, or 5 µg of αDEC205-MSP1_19__PADRE, αDCIR2-MSP1_19__PADRE or ISO-MSP1_19__PADRE and then labeled with anti-IgG-Alexa fluor 488. **(C)** Naïve C57BL/6 splenocytes were incubated with 0.05, 0.5, or 5 µg/mL of each hybrid mAb and stained with fluorescent antibodies. The gating strategy involved the selection of singlets, size versus granulosity and viable cells. Then, CD19^−^CD3^−^CD49b^−^ cells were excluded and CD11c^+^MHCII^+^ dendritic cells (DCs) were gated and subsequently divided in CD8α^+^ and CD8α^−^ DCs. Binding was detected using anti-IgG1-PE antibody. **(B,C)** Analysis was performed using FlowJo software. One experiment representative of three is depicted.

Transfection of HEK293T cells with plasmids encoding the heavy and light chains of the fusion mAbs allowed us to successfully produce and purify αDEC205-MSP1_19__PADRE, αDCIR2-MSP1_19__PADRE and ISO-MSP1_19__PADRE. A schematic representation of the fusion mAbs is depicted in Figure S1 in Supplementary Material. Figure 1A shows a reduced gel in which we observe the heavy (~69 kDa) and light (~25 kDa) chains of all mAbs. To test whether the fusion mAbs maintained their binding capacities to the respective receptors, we performed binding assays using CHO cells constitutively expressing mouse DEC205 or mouse DCIR2 (Figure 1B). We observed that the αDEC205-MSP1_19__PADRE mAb bound specifically, and in a dose dependent manner, to CHO cells expressing exclusively the mouse DEC205 receptor. On the other hand, αDCIR2-MSP1_19__PADRE mAb was able to bind to CHO cells expressing the DCIR2 receptor. As expected, the ISO-MSP1_19__PADRE mAb did not bind to any receptor. To further characterize the binding capacity of the fusion mAbs, we performed a binding assay using splenocytes (Figure 1C). Different concentrations of the fusion mAbs were incubated with C57BL/6 splenocytes. After exclusion of T, B, and NK cells, DC subsets were divided into CD11c^+^MHCII^+^CD8α^+^ or CD11c^+^MHCII^+^CD8α^−^. We observed a dose dependent binding of the αDEC205-MSP1_19__PADRE mAb to the CD8α^+^ DC subset, while the αDCIR2-MSP1_19__PADRE mAb was shown to bind specifically to the CD8α^−^ DC subset. Once more, the ISO-MSP1_19__PADRE mAb did not bind specifically to any DC subset. To verify if the fusion of MSP1_19__PADRE protein to the C-terminal portion of the αDEC205 and αDCIR2 mAbs would affect their binding capacity, we performed an experiment comparing fused and non-fused αDEC205 and αDCIR2 mAbs (Figure S2 in Supplementary Material). We observed that the fusion of the MSP1_19__PADRE protein to αDEC205 and αDCIR2 mAbs did not affect their binding capacity. Taken together, these results led us to conclude that all fusion mAbs were produced successfully and maintained the binding capacity to their respective receptors.

### CpG ODN Promotes Robust Antibody Responses Partially Dependent on TLR9 Signaling after Antigen Targeting to CD8α^+^ or CD8α^−^ DC Subsets

**Figure 2:**
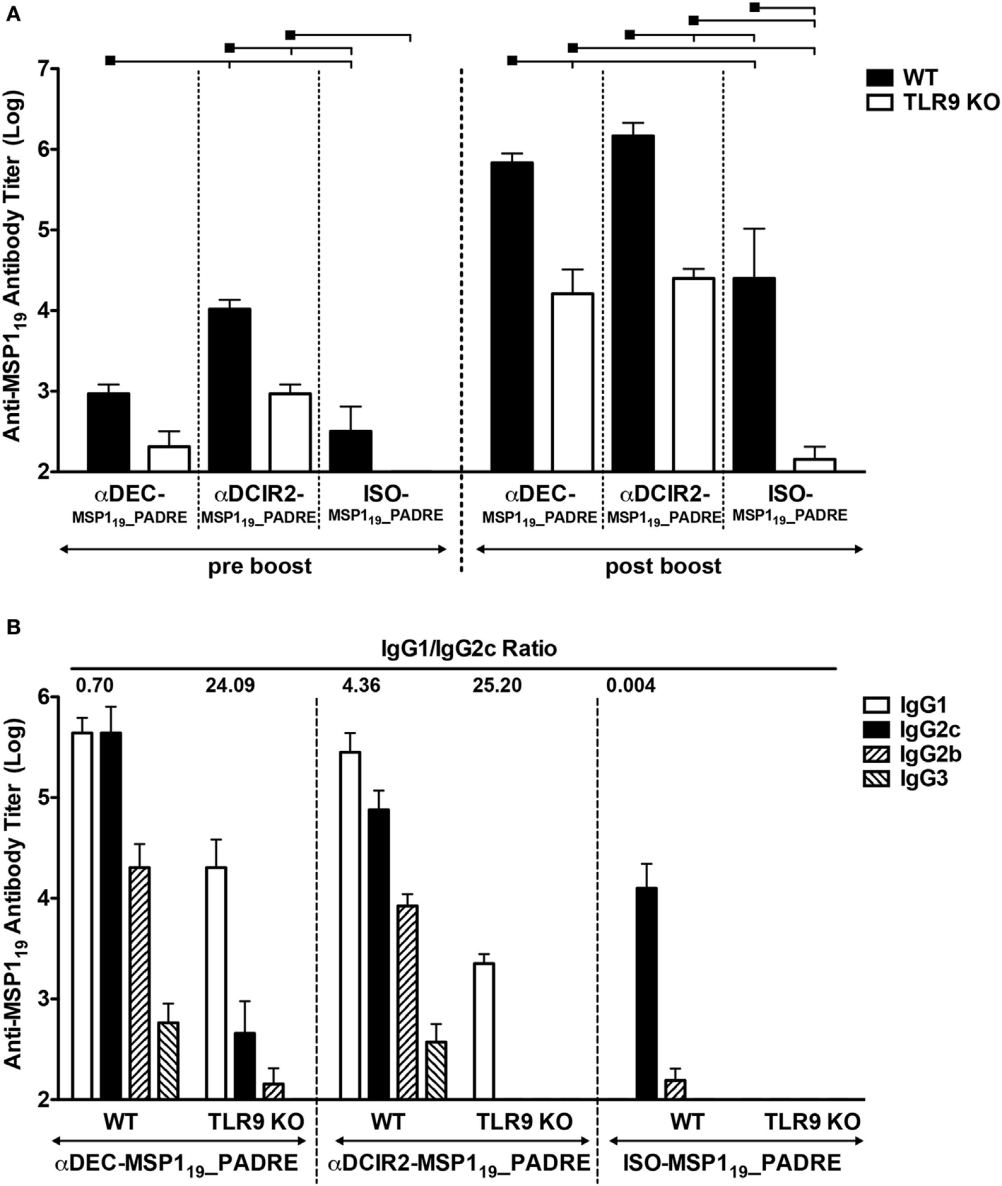
CpG oligodeoxynucleotides (ODN) 1826 as adjuvant induces robust humoral immune response after antigen targeting to CD8α^+^ or CD8α^−^ DC subsets that is partially dependent on TLR9 signaling. WT and TLR9 KO mice were immunized with 5 µg of αDEC205-MSP1_19__PADRE, αDCIR2-MSP1_19__PADRE or ISO-MSP1_19__PADRE together with 25 µg of CpG ODN 1826 as adjuvant. **(A)** Five days before (pre-boost) and 14 days after (post-boost) the administration of the booster dose, blood was collected and serum obtained. Total anti-MSP1_19_ IgG antibodies were detected by ELISA. Graphs show the mean ± SEM of anti-MSP1_19_ titers in different groups normalized in log10 scale (*n* = 5 animals/group). Experiments were analyzed by two-way ANOVA for repeated measures followed by Bonferroni posttest. Black squares indicate the reference group against which comparisons are being made. Horizontal capped lines only depict significant differences (*p* < 0.05). **(B)** Anti-MSP1_19_ IgG1, IgG2b, IgG2c, and IgG3 subclasses were also determined by ELISA 14 days after the boost. The numbers above the bars indicate the IgG1/IgG2c ratio calculated for each group.

To study the role of CpG ODN signaling in the induction of humoral immune response after antigen targeting to CD8α^+^ and CD8α^−^ DC subsets, we immunized wild type (WT) and TLR9 knockout (TLR9 KO) mice with αDEC205-MSP1_19__PADRE, αDCIR2-MSP1_19__PADRE, or with ISO-MSP1_19__PADRE as a non-targeted control. To demonstrate that DCs derived from WT and TLR9KO mice expressed similar amounts of DEC205 or DCIR2 receptors, we stained splenocytes with commercially available αDEC205 (NLDC-145 clone) and αDCIR2 (33D1 clone) mAbs. Figure S3 in Supplementary Material confirms that WT and TLR9KO DCs express similar amounts of DEC205 or DCIR2 receptors. Mice then received two doses of the fusion mAbs in the presence of CpG ODN 1826 and were bled 5 days before (pre-boost) and 14 days after (post-boost) the administration of the second dose (Figure 2). When groups were compared before boost, CD8α^−^ DC targeting through DCIR2 in WT mice induced higher anti-MSP1_19_ antibody titers when compared to targeting through DEC205 or no targeting, indicating that antigen delivery to the CD8α^−^ DC subset induces a more robust primary response. The absence of TLR9 signaling reduced the response in all groups. After the administration of the booster dose, anti-MSP1_19_ antibody titers increased in all groups. Besides, titers were higher (*p* < 0.05) in WT when compared to the TLR9 KO mice, suggesting that CpG ODN 1826 signaling through TLR9 contributes to increase antibody titers after MSP1_19__PADRE targeting to CD8α^+^ or CD8α^−^ DC *via* DEC205 or DCIR2, respectively (Figure 2A). A decrease in anti-MSP1_19_ antibody titers was also observed in mice immunized with the isotype control, indicating that TLR9 signaling also plays a role in the absence of antigen targeting to DCs. Interestingly, after boost, anti-MSP1_19_ antibody titers were not different in mice immunized with αDEC205-MSP1_19__PADRE when compared to animals immunized with αDCIR2-MSP1_19__PADRE, despite the difference observed before the boost. In the absence of antigen targeting (i.e., in animals immunized with the isotype control), anti-MSP1_19_ titers were significantly lower. The same was observed in TLR9 KO mice (Figure 2A).

To study the humoral response in more detail, we also analyzed the anti-MSP1_19_ IgG subclasses elicited after the boost. We observed that WT mice immunized with αDEC205-MSP1_19__PADRE or αDCIR2-MSP1_19__PADRE presented all IgG subclasses tested (IgG1, IgG2b, IgG2c, and IgG3), while ISO-MSP1_19__PADRE immunized WT mice did not present IgG1 antibodies (Figure 2B). Interestingly, we detected differences in the IgG1/IgG2c ratio when WT mice were immunized with αDEC205-MSP1_19__PADRE or with αDCIR2-MSP1_19__PADRE. These differences indicate that antigen targeting, in the presence of CpG ODN 1826, to the CD8α^+^ DCs induced a Th1 prone type of response (IgG1/IgG2c ratio = 0.70), while a more Th2 type of response was induced after antigen targeting to CD8α^−^ DCs (IgG1/IgG2c ratio = 4.36). TLR9 signaling played a role in antibody class switch as we observed a pronounced decrease of IgG2b and 2c in TLR9 KO mice immunized with either αDEC205-MSP1_19__PADRE (IgG1/IgG2c ratio = 24.09) or with αDCIR2-MSP1_19__PADRE (IgG1/IgG2c ratio = 25.20). We did not detect antibody titers after immunization with ISO-MSP1_19__PADRE in the TLR9 KO mice (Figure 2B). Taken together, these results indicate that CpG ODN 1826 increases the humoral immune response when the antigen is targeted to both DC subtypes and that antibody class switch is influenced by TLR9 signaling.

### Antigen Targeting to the CD8α^+^ DC Subset in the Presence of CpG ODN 1826 Elicits Strong CD4^+^ T Cell Response That Is Greatly Diminished in the Absence of TLR9 Signaling

**Figure 3:**
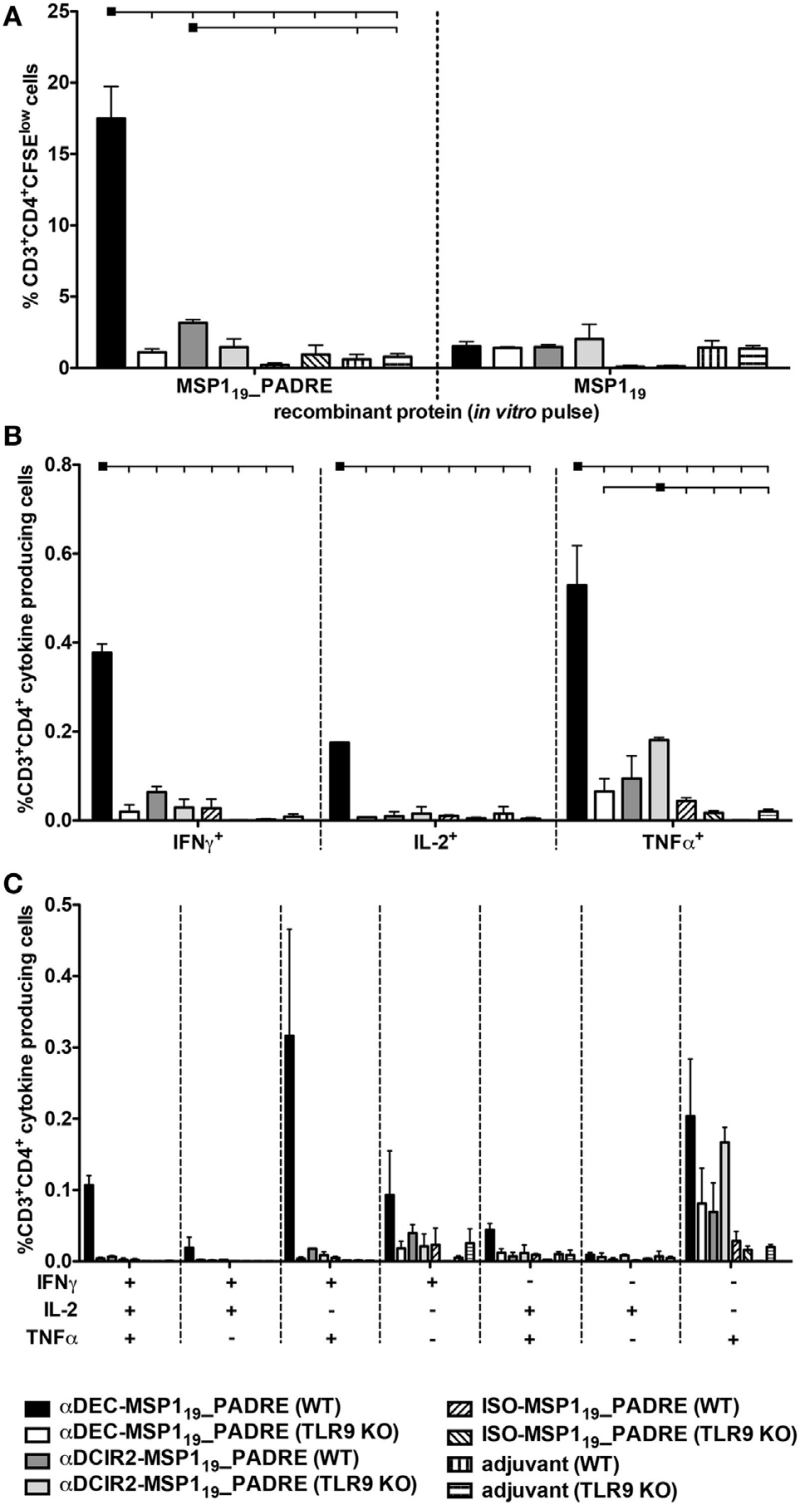
Antigen targeting to the CD8α^+^ dendritic cells (DCs) *via* DEC205 receptor in the presence of CpG oligodeoxynucleotides (ODN) 1826 induces strong CD4^+^ T cell response that is practically abolished in the absence of TLR9 signaling. WT and TLR9 KO mice were immunized with the different hybrid mAbs as described in Figure 2. Twenty days after the administration of the booster dose, mice were euthanized and **(A)** Splenocytes from pooled WT or TLR9 KO mice (*n* = 5) were labeled with CFSE and cultured with 5 µg/mL of either MSP1_19__PADRE or MSP1_19_ recombinant proteins for 96 h. Cells were then stained with fluorescent antibodies, and CD4^+^ T cell proliferation by CFSE dilution was analyzed. The graph shows the percentage of CD3^+^CD4^+^CFSE^low^ T cells after the subtraction of values obtained in the absence of any stimulus. Bars indicate mean ± SEM, and the experiment was analyzed by two-way ANOVA followed by the Bonferroni posttest. **(B)** Splenocytes from pooled mice (*n* = 5 animals/group) were pulsed *ex vivo* with 5 µg/mL of MSP1_19__PADRE recombinant protein and incubated in the presence of brefeldin for 12–16 h. Graphs show the percentage of cells producing IFN-γ, IL-2, or TNFα in the CD3^+^CD4^+^ gate after subtraction of values obtained in the absence of any stimulus. Bars indicate mean ± SEM, and the experiment was analyzed by two-way ANOVA followed by the Bonferroni posttest. Black squares indicate the reference group against which comparisons are being made. Horizontal capped lines only depict significant differences (*p* < 0.05). **(C)** Boolean combinations were created using FlowJo software to determine the frequency of each cytokine production based on all possible combinations. The experiment was performed in duplicates using samples from pooled mice. One representative experiment of two is depicted.

Next, we analyzed the PADRE specific CD4^+^ T cell response in WT and TLR9 KO mice when MSP1_19__PADRE was targeted to either CD8α^+^ or CD8α^−^ DCs (Figure 3). CFSE-labeled splenocytes derived from immunized mice were pulsed *in vitro* with MSP1_19__PADRE or MSP1_19_ recombinant proteins, and after 5 days of culture, the frequency of CD3^+^CD4^+^CFSE^low^ T cells was analyzed by flow cytometry (Figure 3A). A representative gating strategy is depicted in Figure S4 in Supplementary Material. We observed robust CD4^+^ T cell proliferation in WT mice immunized with αDEC205-MSP1_19__PADRE using CpG ODN 1826 as an adjuvant only when cells were pulsed with the recombinant MSP1_19__PADRE. This result was expected since PADRE is an immunodominant peptide and no other peptides, recognized by the C57BL/6 haplotype, have been described in the *P. vivax* MSP1_19_ sequence. In this way, we used the recombinant MSP1_19_ protein pulse as an internal negative control. On the other hand, spleen cells derived from αDEC205-MSP1_19__PADRE immunized TLR9 KO mice showed a very pronounced reduction in proliferation, not different from the one obtained in animals immunized with CpG ODN 1826 only. This result indicates that TLR9 signaling after CpG ODN 1826 stimulation plays a crucial role in the promotion of a CD4^+^ T cell proliferative response after antigen targeting to CD8α^+^ DCs *via* DEC205. In contrast, antigen targeting to CD8α^−^ DCs *via* DCIR2 in the presence of CpG ODN 1826 did not elicit strong specific CD4^+^ T cell proliferation in WT mice. This result was surprising and led us to conclude that, under our experimental conditions, CpG ODN 1826 does not seem to be a good adjuvant to induce CD4^+^ T cell proliferation when the antigen is targeted to the CD8α^−^ DCs *via* DCIR2. We also did not observe proliferation when the ISO-MSP1_19__PADRE mAb was used to immunize WT or TLR9 KO mice.

To further evaluate the PADRE-specific CD4^+^ T cell response, we tested, by intracellular staining, the production of inflammatory cytokines IFN-γ, IL-2, and TNF-α (Figure 3B). Splenocytes from mice immunized with the different fusion mAbs were pulsed with the recombinant MSP1_19__PADRE protein, and intracellular cytokines were labeled after overnight stimulation (representative gating strategy shown in Figure S5 in Supplementary Material). Similarly to what was observed when the CD4^+^ T cell proliferation was analyzed, we detected specific CD4^+^ T cells positive for IFN-γ, IL-2, or TNF-α mainly in αDEC205-MSP1_19__PADRE immunized WT mice. Once more, when TLR9 KO mice were immunized with αDEC205-MSP1_19__PADRE, the frequencies of cytokine-producing cells were negligible. Antigen targeting to CD8α^−^ DCs *via* DCIR2 did not induce specific cells that produced IFN-γ or IL-2. However, we observed a small percentage of TNF-α producing cells in WT or TLR9 KO mice immunized with αDCIR2-MSP1_19__PADRE. In the absence of antigen targeting (when ISO-MSP1_19__PADRE was used), only negligible frequencies of cytokine-producing cells were detected (Figure 3B).

To analyze the cytokine response in more detail, we performed Boolean analysis in order to study cell polyfunctionality. We observed that the specific CD4^+^ T cells produced different combinations of the three cytokines in WT mice immunized with αDEC205-MSP1_19__PADRE, namely IFN-γ^+^IL-2^+^TNF-α^+^, IFN-γ^+^TNF-α^+^, and TNF-α^+^. As expected, immunization of TLR9 KO mice in the same conditions failed to promote an inflammatory response (Figure 3C). Based on these results, we conclude that CpG ODN 1826 stimulation is critical for proliferation and induction of polyfunctional CD4^+^ T cells when the antigen is targeted to CD8α^+^ DCs *via* DEC205. Also, this response is strongly dependent on TLR9 signaling.

### TLR5 Signaling Contributes to Improve the Antibody Response after Priming When the Antigen Is Targeted to CD8α^−^ DCs and after Boosting When the Antigen Is Targeted to CD8α^+^ DCs

**Figure 4:**
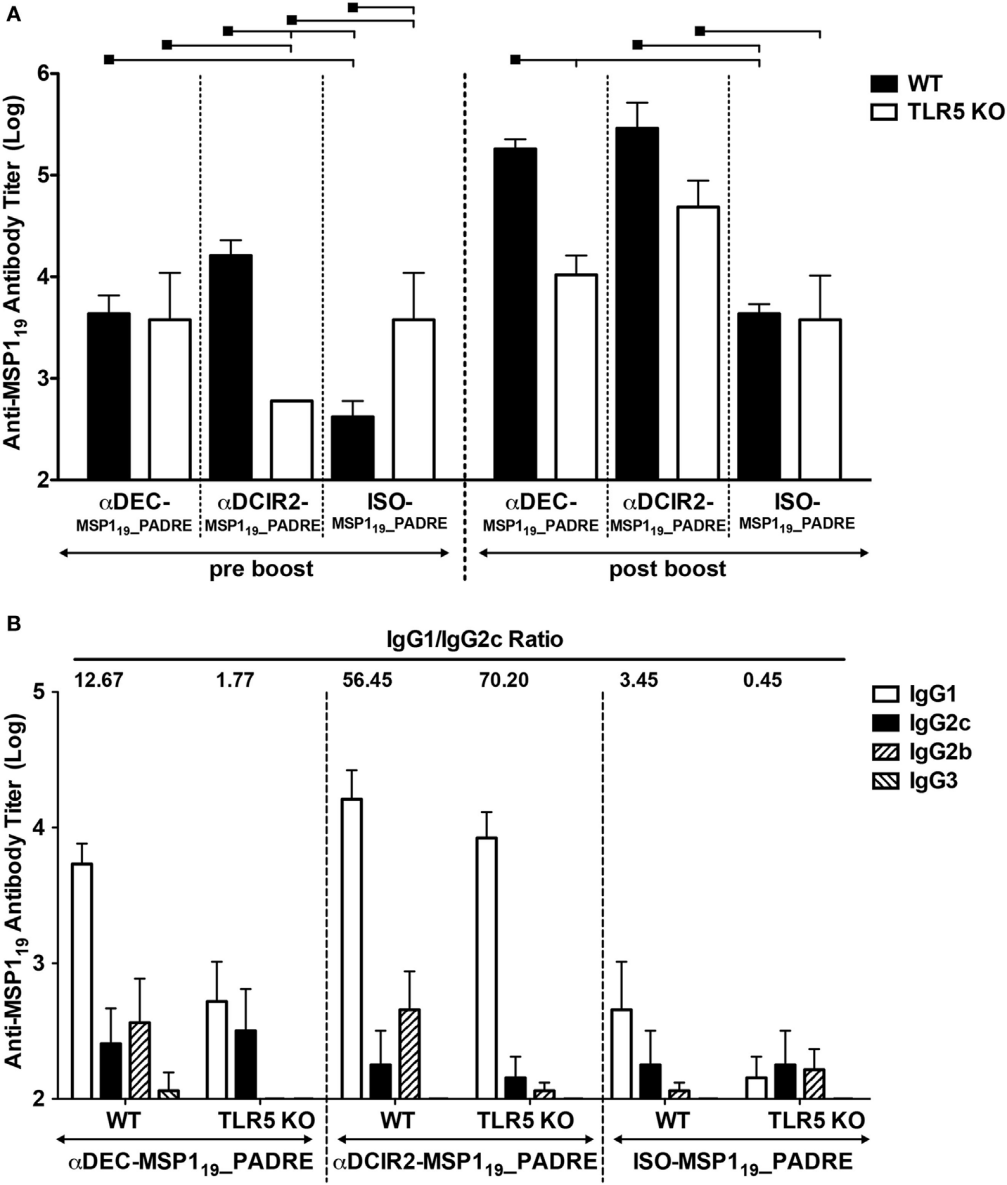
TLR5 signaling contributes after prime or boost, depending on the targeted dendritic cell (DC) subset. WT and TLR5 KO mice were immunized with 5 µg of αDEC205-MSP1_19__PADRE, αDCIR2-MSP1_19__PADRE or ISO-MSP1_19__PADRE together with 5 µg of flagellin as adjuvant. **(A)** Five days before (pre-boost) and 14 days after (post-boost) the administration of the booster dose, blood was collected and serum obtained. Total anti-MSP1_19_ IgG antibodies were detected by ELISA. Graphs show the mean ± SEM of anti-MSP1_19_ titers in different groups normalized in log10 scale (*n* = 5 animals/group). Experiments were analyzed by two-way ANOVA for repeated measures followed by Bonferroni posttest. Black squares indicate the reference group against which comparisons are being made. Horizontal capped lines only depict significant differences (*p* < 0.05). **(B)** Anti-MSP1_19_ IgG1, IgG2b, IgG2c, and IgG3 subclasses were also determined by ELISA 14 days after the boost. The numbers above the bars indicate the IgG1/IgG2c ratio calculated for each group.

To study the contribution of flagellin and TLR5 signaling in the development of a humoral immune response elicited by antigen targeting to CD8α^+^ or CD8α^−^ DCs, groups of WT and TLR5 KO mice were immunized with αDEC205-MSP1_19__PADRE, αDCIR2-MSP1_19__PADRE, or ISO-MSP1_19__PADRE in the presence of recombinant flagellin as adjuvant. It is important to highlight that DCs derived from the TLR5KO mice also expressed similar amounts of DEC205 and DCIR2 receptors when compared to WT (Figure S3 in Supplementary Material). Anti-MSP1_19_ antibody titers were determined before and after the boost. Figure 4A shows that TLR5 signaling is dispensable for antigen targeting to CD8α^+^ DCs before the boost, but it is important if the antigen is directed to CD8α^−^ DCs. In the absence of targeting (i.e., mice immunized with the isotype control), we observed an increase in antibody titers in the absence of TLR5 signaling. After the administration of the booster dose, antibody titers increased in WT mice immunized with all the different fusion mAbs. In TLR5 KO mice, no differences were observed before or after the boost following immunization with αDEC205-MSP1_19__PADRE or ISO-MSP1_19__PADRE. However, in TLR5 KO mice immunized with αDCIR2-MSP1_19__PADRE mAb, the anti-MSP1_19_ titers were increased. When all groups were compared after the boost, we noticed that TLR5 signaling seems to play a role only when CD8α^+^ DCs are targeted *via* DEC205, as we did not observe statistical differences between the WT and TLR5 KO groups immunized with either αDCIR2-MSP1_19__PADRE or ISO-MSP1_19__PADRE. In summary, in the presence of flagellin and after the second dose, DC targeting to both DC subsets leads to an increased humoral response in comparison with the absence of targeting.

Moreover, anti-MSP1_19_ IgG1, IgG2b, IgG2c, and IgG3 subclasses were determined by ELISA after boost. All groups, except the TLR5 KO mice immunized with αDEC205-MSP1_19__PADRE, presented detectable titers of IgG1, IgG2b, and IgG2c. Very low (or undetectable) levels of IgG3 titers were also detected. Contrary to what was observed in the WT animals immunized with CpG ODN 1826, mice immunized with flagellin did not promote vigorous class switch as the IgG1/IgG2c ratio was higher than 1 in all groups, except in the TLR5 KO mice immunized with ISO-MSP1_19__PADRE (Figure 4B). Interestingly, WT and TLR5 KO mice immunized with αDCIR2-MSP1_19__PADRE presented high IgG1/IgG2c ratios (56.45 and 70.20, respectively), while in mice immunized with αDEC205-MSP1_19__PADRE these ratios were much lower (12.67 and 1.77, respectively). Of note, IgG2b titers were drastically reduced in the absence of TLR5 signaling when the antigen was targeted to both DC subsets. This result indicates that TLR5 signaling influences class switch.

### Flagellin Is Important for the Induction of CD4^+^ T Cell Proliferation but Not for the Production of Inflammatory Cytokines When CD8α^−^ DCs Are Targeted *via* DCIR2

**Figure 5:**
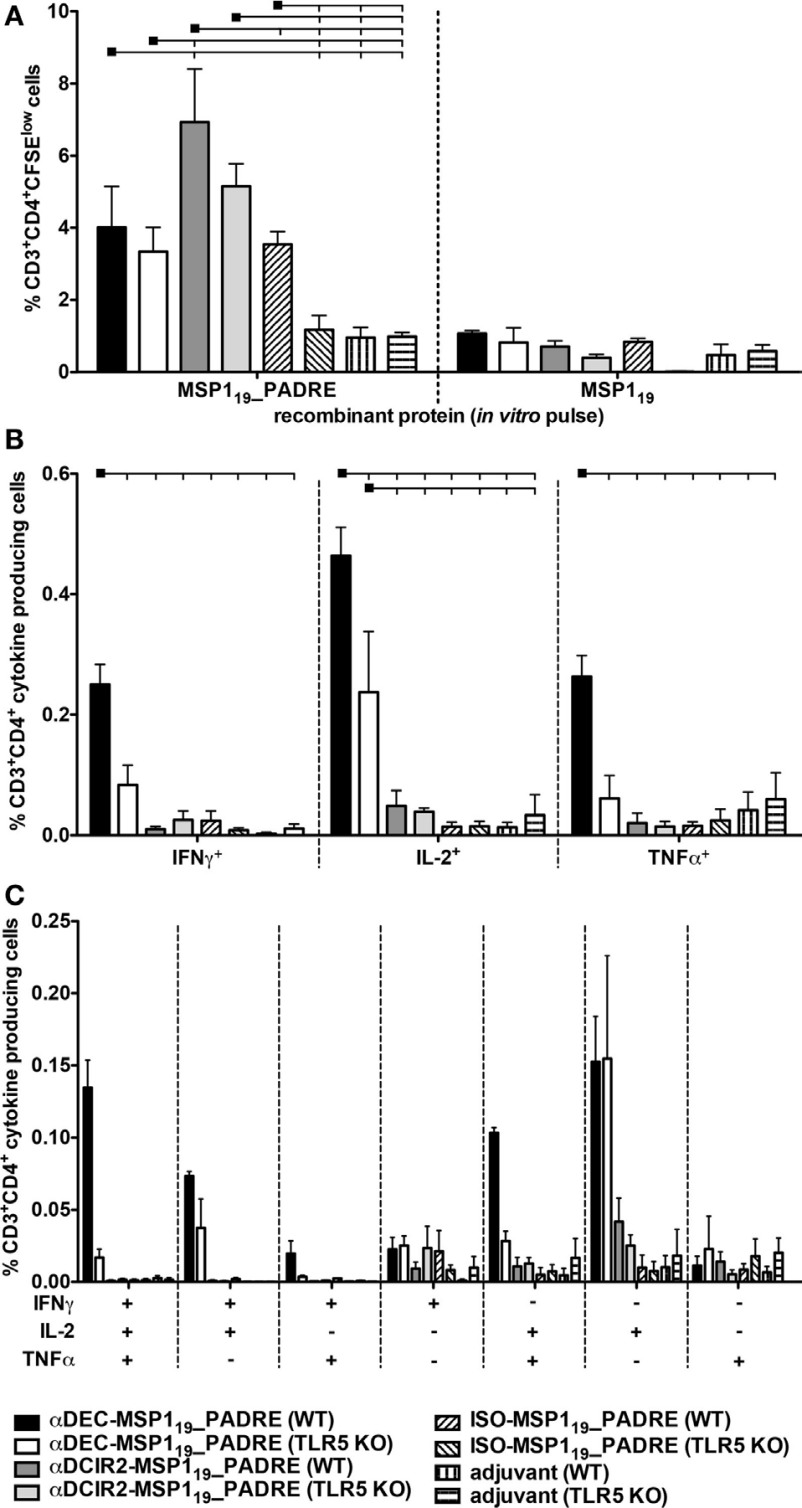
Antigen targeting to CD8α^−^ dendritic cells (DCs) *via* DCIR2 in the presence of flagellin induces CD4^+^ T cell proliferation but no production of pro-inflammatory polyfunctional CD4^+^ T cells. WT and TLR5 KO mice were immunized with the different hybrid monoclonal antibodies (mAbs) as described in Figure 4. Twenty days after the administration of the booster dose, mice were euthanized and **(A)** Splenocytes from pooled WT or TLR5 KO mice (*n* = 5) were labeled with CFSE and cultured with 5 µg/mL of either MSP1_19__PADRE or MSP1_19_ recombinant proteins for 96 h. Cells were then stained with fluorescent antibodies, and CD4^+^ T cell proliferation was analyzed by CFSE dilution. The graph shows the percentage of CD3^+^CD4^+^CFSE^low^ T cells after the subtraction of values obtained in the absence of any stimulus. Bars indicate mean ± SEM, and the experiment was analyzed by two-way ANOVA followed by the Bonferroni posttest. **(B)** Splenocytes from pooled mice (*n* = 5 animals/group) were pulsed *ex vivo* with 5 µg/mL of MSP1_19__PADRE recombinant protein and incubated in the presence of brefeldin for 12–16 h. Graphs show the percentage of cells producing IFN-γ, IL-2, or TNFα in the CD3^+^CD4^+^ gate after subtraction of values obtained in the absence of any stimulus. Bars indicate mean ± SEM, and the experiment was analyzed by two-way ANOVA followed by the Bonferroni posttest. Black squares indicate the reference group against which comparisons are being made. Horizontal capped lines only depict significant differences (*p* < 0.05). **(C)** Boolean combinations were created using FlowJo software to determine the frequency of each cytokine production based on all possible combinations. The experiment was performed in duplicates using samples from pooled mice. One representative experiment of two is depicted.

We next analyzed the CD4^+^ T cell proliferation elicited when MSP1_19__PADRE was targeted to both DC subsets in the presence of flagellin (representative gating strategy depicted in Figure S4 in Supplementary Material). We observed higher CD4^+^ T cell proliferation in WT mice immunized with αDCIR2-MSP1_19__PADRE when compared to the groups immunized with αDEC205-MSP1_19__PADRE or ISO-MSP1_19__PADRE. Once more, MSP1_19_ recombinant protein was used as a negative control, and we did not observe significant proliferation among all the groups. Interestingly, for αDEC205-MSP1_19__PADRE or αDCIR2-MSP1_19__PADRE mAbs, despite a reduction, proliferation does not seem to depend on TLR5 signaling, as we did not observe statistically significant differences when we compared WT with TLR5 KO mice. On the other hand, TLR5 signaling seems important in the absence of targeting (i.e., in mice immunized with ISO-MSP1_19__PADRE, Figure 5A). These results indicate that flagellin is important for the induction of CD4^+^ T cell proliferation when CD8α^−^ DCs are targeted *via* DCIR2. However, TLR5 signaling does not seem to play a major role in the CD4^+^ T cell proliferation when the antigen is delivered to either CD8α^+^ or CD8α^−^ DCs *via* DEC205 or DCIR2, respectively.

Surprisingly, when the frequency of CD4^+^ T cells producing inflammatory cytokines (IFN-γ, IL-2, and TNF-α) was analyzed (a representative gating strategy is depicted in Figure S5 in Supplementary Material), we did not detect many cells producing any of these cytokines in the WT or TLR5 KO groups immunized with αDCIR2-MSP1_19__PADRE. On the other hand, specific CD4^+^ T cells producing IFN-γ, IL-2, or TNF-α were detected in the WT group immunized with αDEC205-MSP1_19__PADRE. This response was reduced in TLR5 KO mice (Figure 5B). Similar results were obtained when polyfunctional CD4^+^ T cells were analyzed (Figure 5C). We conclude that antigen targeting to CD8α^−^ DCs *via* DCIR2 in the presence of flagellin induces CD4^+^ T cell proliferation. However, induction of inflammatory polyfunctional CD4^+^ T cells is only observed when the antigen is targeted specifically to the CD8α^+^ DCs *via* DEC205 and is partially dependent on TLR5 signaling.

In an attempt to verify if other cytokines were being produced, we analyzed the production of IL-4, IL-6, IL-17A, and IL-10 in the supernatant of cell cultures, 96 h after pulse, using recombinant MSP1_19__PADRE or MSP1_19_ proteins in WT mice immunized with the different fusion mAbs (Figure S6 in Supplementary Material). We detected higher levels of IL-4 and IL-10 in WT mice immunized with αDCIR2-MSP1_19__PADRE when compared to mice immunized with αDEC205-MSP1_19__PADRE together with CpG ODN 1826 (Figures S6A,B in Supplementary Material, respectively) or flagellin (Figures S6C,D in Supplementary Material, respectively). The production of IL-6 and IL-17A was below the kit detection threshold (data not shown). Taken together, these results suggest that antigen targeting to CD8α^−^ DCs *via* DCIR2 in the presence of CpG ODN 1826 or flagellin induces more Th2/regulatory response.

### Differential Expression of Co-stimulatory Molecules in CD8α^+^ and CD8α^−^ DCs Induced by CpG ODN 1826 or Flagellin

Due to differences in CD4^+^ T cell proliferation induced by antigen targeting to CD8α^+^ and CD8α^−^ DCs using CpG ODN 1826 or flagellin as adjuvants, we hypothesized that CD8α^+^ and CD8α^−^ DCs may differently respond to these activation stimuli. CD8α^+^ DC targeting *via* DEC205 induced Th1 CD4^+^ T cell polarization when CpG ODN 1826 or flagellin were used. On the other hand, CD8α^−^ DC targeting using the same adjuvants induced more IL-4 and IL-10 in culture supernatants, and robust CD4^+^ T cell proliferation when flagellin was used.

To further gain insight into those differences, we sorted spleen CD8α^+^ and CD8α^−^ DCs. After isolation, both DC subsets were stimulated with CpG ODN 1826 or flagellin for 48 h. Negative controls were left untreated. Then, TNFα and IL-6 in culture supernatants were measured (Figure S7 in Supplementary Material). DC stimulation with CpG ODN 1826 induced TNFα and IL-6 production in both DC subsets. However, CD8α^−^ DCs were much more responsive and produced approximately 7 times more TNFα or 13 times more IL-6. When flagellin was used as adjuvant, CD8α^+^ DCs did not secrete TNFα or IL-6. On the other hand, CD8α^−^ DCs secreted more TNFα or IL-6, although the latter difference was not statistically significant (when compared to non-stimulated DCs). These results indicate that CpG ODN 1826 is able to directly activate CD8α^−^ DCs to produce more TNFα or IL-6 when compared to CD8α^+^ DCs, while flagellin only directly activates CD8α^−^ DCs.

**Figure 6:**
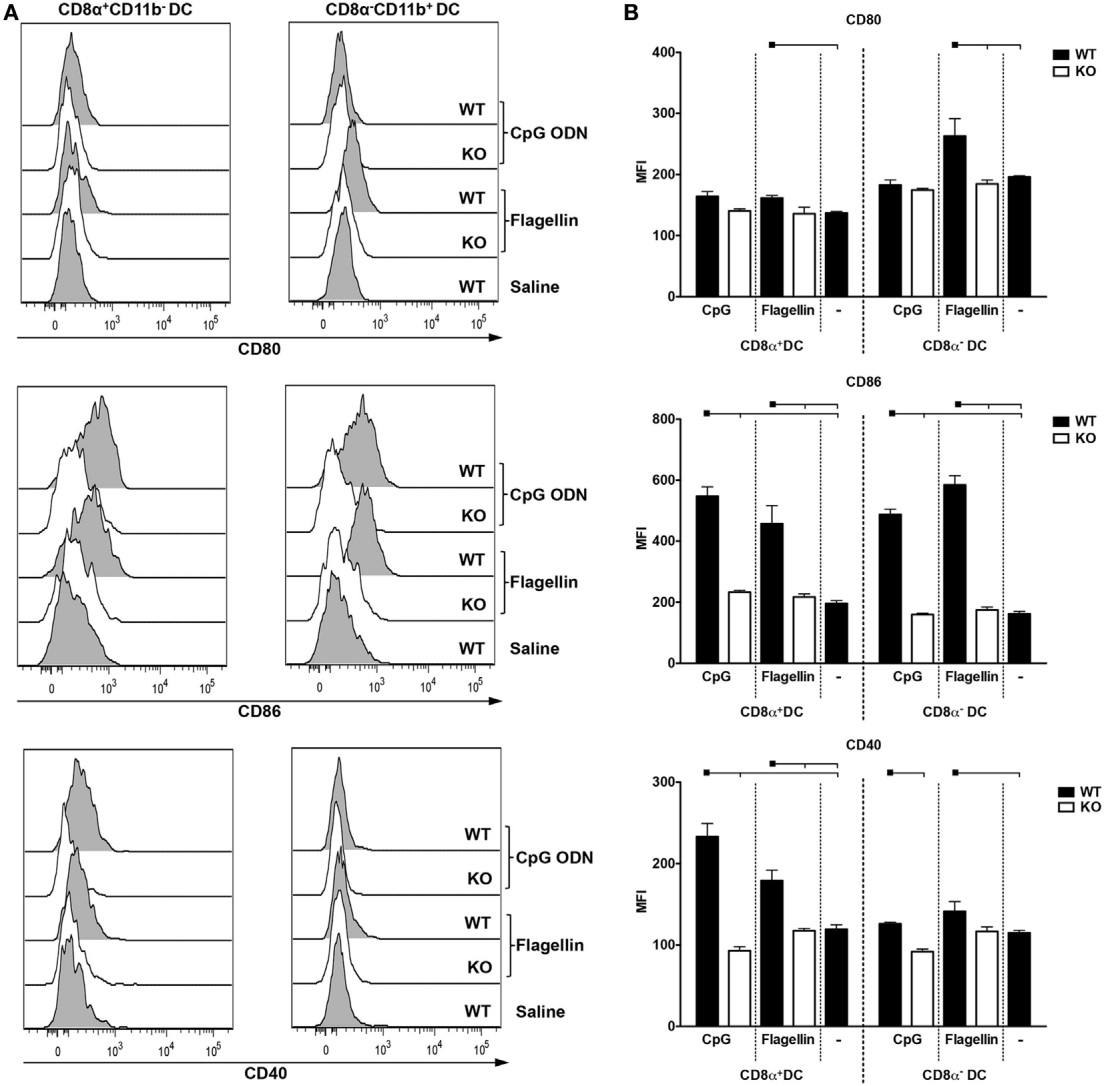
CpG oligodeoxynucleotides (ODN) 1826 or flagellin induce differential expression of co-stimulatory molecules in CD8α^+^ and CD8α^−^ dendritic cells (DCs). C57BL/6 naive mice were injected i.p. with 25 µg of CpG ODN 1826 or with 5 µg of flagellin. 6 h later, mice were euthanized and splenocytes were stained with different fluorescent antibodies. The gating strategy is depicted in Figure S8 in Supplementary Material. **(A)** Representative histograms showing the expression of the co-stimulatory molecules CD80, CD86, and CD40 on CD8α^+^ and CD8α^−^ DCs. **(B)** Graphs show the mean ± SEM of the median fluorescence intensity for CD80, CD86, and CD40 obtained on CD8α^+^CD11b^−^ and CD8α^−^CD11b^+^ DCs from three mice per group. The experiment was analyzed by two-way ANOVA followed by the Bonferroni posttest. Black squares indicate the reference group against which comparisons are being made. Horizontal capped lines only depict significant differences (*p* < 0.05). One representative experiment of three is depicted.

We then decided to investigate DC subset expression of co-stimulatory molecules after *in vivo* administration of CpG ODN 1826 or flagellin to WT and KO mice. As negative controls, we used WT mice immunized with saline. We analyzed the upregulation of CD80, CD86, and CD40 on CD8α^+^CD11b^−^ (DEC205^+^) and CD8α^−^CD11b^+^ (DCIR2^+^) DC subtypes according to the gating strategy depicted in Figure S8 in Supplementary Material. 6 h after injection, we observed a significant increase in the median fluorescence intensity (MFI) of CD86 and CD40 in both DC subsets in WT mice immunized with CpG ODN 1826 when compared to saline. This increase was reverted in TLR9 KO mice (Figures 6A–B, middle and lower panels). Although we observed a slight increase in CD80 expression, especially in CD8α^+^CD11b^−^ DCs when compared to saline or TLR9 KO mice, the difference was not statistically significant (Figures 6A–B, upper panels). When we analyzed DCs derived from mice immunized with flagellin, we observed an increase in CD80, CD86, and CD40 MFIs in both DC subtypes when compared to saline. The absence of TLR5 signaling also impaired MFI upregulation (Figure 6, all panels). We conclude that either CpG ODN 1826 or flagellin administration induces significant upregulation of co-stimulatory molecules in both DC subsets *in vivo* after 6 h of inoculation.

## Discussion

Antigen targeting to DCs through DEC205 and DCIR2 receptors is a largely used strategy to induce specific immune responses to antigens. As previously described, the use of an adjuvant is required to promote a non-tolerogenic immune response (12, 25). Here, we studied the immune responses induced by MSP1_19__PADRE antigen targeting to CD8α^+^ and CD8α^−^ DCs *via* DEC205 and DCIR2 receptors using CpG ODN 1826 or flagellin as adjuvants. First, we successfully produced the fusion mAbs αDEC205- MSP1_19__PADRE, αDCIR2- MSP1_19__PADRE, and the isotype control (ISO-MSP1_19__PADRE). MSP1_19__PADRE is a chimeric antigen designed to increase MSP1_19_ antigenicity. Our immunization results confirmed that PADRE epitope elicited robust cellular immune responses while MSP1_19_ induced high antibody titers as previously described (43). We showed that CpG ODN 1826 and flagellin were efficient to induce antibody production, proliferation, and pro-inflammatory CD4^+^ T cell responses when MSP1_19__PADRE was targeted to CD8α^+^ DCs *via* DEC205. However, when the CD8α^−^ DCs were targeted, different outcomes were observed. In CpG ODN 1826 immunized mice, we observed an increase in antibody responses, and the development of a more Th2 type of response, corroborated by the increase in IL-4 production. On the other hand, when we analyzed CD4^+^ T cell proliferation or pro-inflammatory cytokine production, the response was negligible. An interesting observation was made when flagellin was used as adjuvant. In this case, we observed CD4^+^ T cell proliferation but no induction of pro-inflammatory cytokines. Again, we detected an increase in IL-4 production. These results led us to conclude that each adjuvant seemed to differentially influence the promotion of adaptive immune responses when the antigen was targeted to CD8α^+^ and CD8α^−^ DCs.

CpG oligodeoxynucleotides (ODN) 1826 is a TLR9 ligand expressed in antigen-presenting cells, including DCs and B cells (31). It can stimulate activated B cells by direct TLR9 signaling and promote their differentiation into plasma cells. Also, antigen-experienced B cells upregulate TLR9 and can be activated by CpG ODN 1826, increasing antibody production (50–52). In our system, the use of CpG ODN 1826 as an adjuvant induced high antibody titers when mice were immunized with either αDEC205-MSP1_19__PADRE or αDCIR2-MSP1_19__PADRE. These titers were significantly reduced in the absence of TLR9 signaling. Our results also indicated that TLR9 signaling *via* CpG ODN 1826 influences antibody class switch, promoting IgG2b and mainly IgG2c subclasses when mice are immunized with αDEC205-MSP1_19__PADRE. Immunization with αDCIR2-MSP1_19__PADRE showed an even more pronounced effect as class switch to IgG2b and IgG2c was completely abolished in TLR9 KO mice. A possible explanation for this effect may be related to the fact that CpG ODN 1826 increases germinal center reaction induced by helper T cells primed by matured DC, supporting class switch to IgG2b and IgG2c subclasses (53). In fact, it was previously shown that CD8α^−^ DCs are known to induce functional antigen-specific Tfh cells that play a central role in antibody production (41, 54). It is interesting to mention that in the absence of antigen targeting, CpG ODN 1826 signaling also played a crucial role in antibody production and class switch.

A different set of results was obtained when flagellin was used as adjuvant. First, TLR5 signaling was only partially important for the induction of antibodies when CD8α^+^ DCs were targeted. Antigen targeting to CD8α^−^ DCs, or absence of targeting, were not influenced by flagellin signaling through TLR5. Class switching was mainly restricted to IgG1 and not influenced by the absence of TLR5 signaling, mainly when the antigen was delivered through αDCIR2-MSP1_19__PADRE. The effect of flagellin in inducing a Th2 type of response with the production of high levels of specific IgG1 was previously reported (55, 56). The partial effect observed in TLR5 KO mice may also be explained by the fact that, once intracellular, flagellin is able to signalize through Naip5/NLRC4 inflammasome (32, 34, 57), and induce DC activation. Furthermore, there are data indicating that flagellin can stimulate antibody production in a TLR5 and NAIP5 independent fashion (56).

Interesting results were also obtained when we analyzed the proliferation of specific CD4^+^ T cells when the antigen was targeted to CD8α^+^ and CD8α^−^ DCs in the presence of CpG ODN 1826 or flagellin. When CpG ODN 1826 was used as adjuvant, a very pronounced T cell proliferation was only observed in WT mice immunized with αDEC205-MSP1_19__PADRE. This response was almost completely abolished in the absence of TLR9 signaling. More interesting was the result obtained when the CD8α^−^ DCs were targeted *via* DCIR2. In this case, we were unable to detect specific proliferation in WT or TLR9 KO mice, indicating that antigen delivery to this particular DC subset in the presence of CpG ODN 1826 is not an efficient way to induce CD4^+^ T cell proliferation under our experimental conditions. This result contrasts with reports that observed vigorous CD4^+^ T cell proliferation after antigen targeting to the CD8α^−^ DCs (12, 58). This difference may be explained by differences in the immunization protocols and/or in the adjuvants used. While both reports used transgenic T cell transference and analyzed proliferation 3 or 9 days after the administration of one dose of the chimeric mAbs, or *in vitro*, we administered two doses of each mAb and analyzed the CD4^+^ T cell immune response 20 days after the boost. Also, both authors used either the agonist αCD40 mAb or a combination of αCD40 mAb plus poly (I:C). Another important point is that, as mentioned before, the CD8α^−^ DC subset is very efficient to induce Tfh cells (41, 54) that may not necessarily present strong proliferation capacity. On the other hand, when flagellin was used, we detected specific T cell proliferation in response to antigen targeting especially to CD8α^−^ DCs, result that agrees with previous reports (12, 58). Despite a reduction, the response obtained in the absence of TLR5 signaling was not significantly different from that obtained in its presence. Furthermore, antigen targeting to the CD8α^+^ DCs induced a lower level of CD4^+^ T cell proliferation in the presence or absence of TLR5 signaling. In summary, TLR5 direct signaling seems dispensable for the induction of antigen-specific CD4^+^ T cell proliferation after antigen targeting to CD8α^+^ or CD8α^−^ DC subsets.

When we analyzed the induction of specific CD4^+^ T cells that produced pro-inflammatory cytokines, we noticed that the response was mainly dependent on the targeted DC subset. The CD4^+^ T cells response was similar when the antigen was targeted to CD8α^+^ DCs *via* DEC205 using either CpG ODN 1826 or flagellin as adjuvants. Immunizations with αDEC205-MSP1_19__PADRE in the presence of CpG ODN 1826 or flagellin induced polyfunctional IFN-γ^+^IL-2^+^TNFα^+^ CD4^+^ T cells. Antigen targeting to CD8α^+^ DCs *via* DEC205 also induced inflammatory cytokines in the presence of poly (I:C), a TLR3/MDA5 ligand (27–29, 59). Taken together, these results confirm that antigen targeting to CD8α^+^ DCs is independent of the adjuvant but dependent of DC subtype. On the contrary, very low percentages of pro-inflammatory cytokine-producing cells were obtained when the antigen was targeted to CD8α^−^ DCs using either CpG ODN 1826 or flagellin, while higher levels of IL-4 and IL-10 were detected in culture supernatants. Detection of IL-4 in culture supernatants was previously reported when CD8α^−^ DCs were targeted *via* DCIR2 (58). This lack of pro-inflammatory cytokine production when the antigen is delivered through DCIR2 may also be explained by the fact that CD8α^−^ DCs are specialized in antigen presentation and induction of Tfh cells (41, 54). In this way, it is plausible to speculate that they may not induce the activation of Th1 cells capable of producing IFN-γ, IL-2, and TNFα.

Up to this point, our results suggested that the adjuvants might help in the development of humoral immune responses, while it is the DC subset that essentially dictates the fate of the CD4^+^ T cell response. To explore in more detail DC subset activation by the two adjuvants, we performed experiments *in vitro* and *in vivo*. Purified splenic WT DCs were incubated with either CpG ODN 1826 or flagellin, and TNFα or IL-6 secretion was detected 48 h later. We observed that CpG ODN 1826 was able to induce cytokine production by both DC subsets while flagellin only activated the CD8α^−^ DCs. In *in vivo* experiments, we administered CpG ODN 1826 or flagellin to WT or KO mice, and 6 h later analyzed the upregulation of co-stimulatory molecules. CpG ODN 1826 induced mainly upregulation of CD86 and CD40 in both DC subsets. Previous reports showed that both DCs subsets are in fact able to respond to CpG ODN as they express similar levels of TLR9 transcripts (60), and also upregulate co-stimulatory molecules (61). Interesting results were obtained when flagellin was used *in vitro* and *in vivo*. In this case, flagellin was not able to directly activate CD8α^+^ DCs. This can be explained by the fact that this particular subset does not express TLR5 (60). However, an upregulation in co-stimulatory molecules was observed *in vivo*. Previous reports also show conflicting results when flagellin was used. Some investigators showed direct activation of murine bone marrow-derived DCs (55, 62, 63), while others reported an effect on human, but not murine, DCs (64). Salazar-Gonzalez et al. obtained similar results to ours when flagellin was administered to mice, but no effect when flagellin was added directly to purified DCs. In this way, they suggested that the stimulatory effect of flagellin on splenic DCs is indirect (65).

In summary, our results indicate that the combination of CpG ODN 1826 and flagellin with antigen delivery to the two major conventional DC subsets induces different effects on the humoral and cellular immune responses. While both adjuvants are efficient to induce Th1 responses when the antigen is directed to CD8α^+^ DCs, a more Th2/Treg type of response is obtained when the antigen is directed to the CD8α^−^ DCs. This knowledge may be explored for the design of DC-targeted vaccines aiming to use CpG ODN 1826 or flagellin as adjuvants. The best combination of antigen targeting/adjuvant will depend mainly on the correlates of protection for a given disease.

## Ethics Statement

All experimental procedures and animal handling were performed in accordance with the National Institutes of Health Guide for the Care and Use of Laboratory Animals and with the Brazilian National Law on animal care (11.794/2008). The Institutional Animal Care and Use Committee (CEUA) of the University of São Paulo approved all procedures under the protocol number 082.

## Author Contributions

RA, FS, and SB designed the experiments. RA, FS, KA, BA, NF, and MY conducted most of the experiments. RA, FS, and SB analyzed the data. FS and SB prepared the figures and wrote the manuscript. IS, LF, and DR contributed reagents. All authors read and approved the final version of the manuscript.

## Conflict of Interest Statement

The authors declare that the research was conducted in the absence of any commercial or financial relationships that could be construed as a potential conflict of interest.
