# Supplementary material for: CpG Oligodeoxinucleotides and Flagellin Modulate the Immune Response to Antigens Targeted to CD8α+ and CD8α− Conventional Dendritic Cell Subsets
Source: Front Immunol. 2017 Dec 4;8:1727. doi: 10.3389/fimmu.2017.01727 (PMC5723008; doi:10.3389/fimmu.2017.01727)
Supplement: Supplementary file 1 [file Data_Sheet_1.docx]

Supplementary Material

**CpG oligodeoxinucleotides and flagellin modulate the immune response to antigens targeted to CD8α^+^ and CD8α^-^ conventional dendritic cell subsets.**

Renan Antonialli^1¶^, Fernando B. Sulczewski^1¶^, Kelly Nazaré da Silva Amorim^1^, Bianca da Silva Almeida^1^, Natália Soares Ferreira^1^, Márcio Massao Yamamoto^1^, Irene da Silva Soares^2^, Luís Carlos de Souza Ferreira^3^, Daniela Santoro Rosa^4,5^, Silvia Beatriz Boscardin^1,5^*

^1^ Department of Parasitology, Institute of Biomedical Sciences, University of São Paulo, São Paulo, Brazil

^2^ Department of Clinical and Toxicological Analysis, School of Pharmaceutical Sciences, University of São Paulo, São Paulo, Brazil

^3^ Department of Microbiology, Institute of Biomedical Sciences, University of São Paulo, São Paulo, Brazil

^4^ Department of Microbiology, Immunology and Parasitology, Federal University of São Paulo, São Paulo, Brazil

^4^ Institute for Investigation in Immunology (iii), INCT, São Paulo, Brazil

* Correspondence:

Dr. Silvia Beatriz Boscardin

sbboscardin@usp.br

## Supplementary Figures

**Supplementary Figure 1. Schematic representation of the hybrid monoclonal antibodies.** Heavy chain variable region (V_H_), light chain (kappa) variable region (V_k_), constant light (kappa) chain (C_k_), and constant heavy chain (C_H_).

**Supplementary Figure 2. Fusion of the MSP1_19__PADRE protein to the C-terminal portion of the αDEC205 and αDCIR2 mAbs does not affect their binding capacity.** Naïve C57BL/6 splenocytes were incubated with 0.1, 1 or 10 μg/mL of each hybrid mAb or with the respective non-fused mAbs, and stained with fluorescent antibodies. The gating strategy is depicted in Figure 1C. Histograms show αDEC205-MSP1_19_PADRE and αDEC205 binding to the CD8α^+^ DCs **(A)** or αDCIR2-MSP1_19_PADRE or αDCIR2 binding to the CD8α^-^ DCs **(B)**. Analysis was performed using FlowJo software.

**Supplementary Figure 3. WT, TLR5KO and TLR9KO DCs express similar amounts of DEC205 and DCIR2 receptors.** C57BL/6 WT, TLR5KO and TLR9 KO naive mice were euthanized and splenocytes were stained with different fluorescent antibodies. The gating strategy is depicted in Supplementary Figure 8. Histograms show CD8α^+^CD11b^-^ DCs from each group (WT, TLR5KO or TLR9KO) stained with the rat NLDC-145 mAb (left panel) while the CD8α^-^CD11b^+^ DCs were stained with the rat 33D1 mAb (right panel). Control in the left panel was gated on CD8α^-^CD11b^+^ DCs (DCIR2 expressing DCs), while in the right panel the gate was made in the CD8α^+^CD11b^-^ DCs (DEC205 expressing DCs). Analysis was performed using FlowJo software.

**Supplementary Figure 4. Representative dot plots of the gating strategy used for the analysis of CD4^+^ T cell proliferation.** WT and KO naive mice were immunized with the different hybrid mAbs as described in Figures 2 and 4. Twenty days after the administration of the booster dose, mice were euthanized, total splenocytes were stained with 1.25 μM CFDA dye, and cultured in the presence or absence of recombinant MSP1_19__PADRE and MSP1_19_ proteins for 96 hours. Cells were stained with different fluorescent antibodies. The gating strategy consisted of gates on singlets followed by size x granulosity, CD3^+^CD4^+^, and finally CD4^+^CFSE^low^. Analysis was performed using FlowJo software.

**Supplementary Figure 5. Representative dot plots of the gating strategy used for the analysis of the intracellular cytokine production.** WT and KO naive mice were immunized with the different hybrid mAbs as described in Figures 2 and 4. Twenty days after the administration of the booster dose, mice were euthanized, and total splenocytes were pulsed *ex vivo* with 1μg/mL of MSP1_19__PADRE recombinant protein and incubated in the presence of brefeldin for 12-16 hours. Cells were stained with different fluorescent antibodies. The gating strategy consisted of gates on singlets followed by size x granulosity, CD3^+^, CD4^+^, and finally CD4^+^IFN-γ, CD4^+^IL-2^+^ or CD4^+^TNFα^+^. Analysis was performed using FlowJo software.

**Supplementary Figure 6. IL-4 and IL-10 production was higher after antigen targeting to CD8α^-^ DCs in the presence of CpG ODN or flagellin.** C57BL/6 mice were immunized with the different hybrid mAbs in the presence of 25 μg of CpG **(A and B)** or 5 μg of flagellin **(C and D)**. Twenty days after the boost, mice were euthanized and the splenocytes were incubated with 1 μg/mL of the recombinant MSP1_19__PADRE or MSP1_19_ proteins. Ninety-six hours later supernatants were collected, and IL-4 **(A and C)** and IL-10 **(B and D)** levels were detected by cytometric bead arrays. Graphs show the concentration (pg/mL) of IL-4 and IL-10 after subtraction of values obtained in the absence of any stimulus. Bars indicate mean ± SEM of pooled groups in triplicates. The experiment was analyzed by two-way ANOVA followed by the Bonferroni post-test. Horizontal capped lines represent p-values < 0.05.

**Supplementary Figure 7. TNFα and IL-6 production in CD8α^+^ and CD8α^-^ DCs in the presence of CpG ODN 1826 or flagellin.** Splenocytes from C57BL/6 mice were submitted to a low density gradient of BSA, stained and then CD8α^+^ and CD8α^-^ DCs were sorted. DC subtypes were stimulated *in vitro* with 32 μg/mL CpG ODN 1826 or with 1 μg/mL flagellin for 48 hours. As negative controls, DC subsets were left untreated. **(A)** TNFα or **(B)** IL-6 concentrations were measured by Cytometric Bead Array. Graphs show the mean ± SEM of the concentration detected in culture supernatants. The experiment was analyzed by two-way ANOVA followed by the Bonferroni post-test. Horizontal capped lines represent p-values < 0.05.

**Supplementary Figure 8. Representative dot plots of the flow cytometry panel used for the analysis of the co-stimulatory molecules CD80, CD86 and CD40.** C57BL/6 naive mice were injected i.p. with 25 μg of CpG ODN or with 5 μg of flagellin. Six hours later, mice were euthanized and splenocytes were stained with different fluorescent antibodies. The gating strategy consisted of gates on singlets followed by size x granulosity, live cells, CD3^-^CD19^-^CD49b^-^MHCII^+^, CD11c^+^ and finally CD8α^-^CD11b^+^ (DCIR2 expressing cells) or CD8α^+^CD11b^-^ (DEC205 expressing cells). These gatings were used to calculate the median fluorescence intensities (MFIs) shown in Figure 6.

**Supplementary Materials and Methods**

**Detection of cytokines by cytometric bead array (CBA)**

Splenocytes (1x10^6^ cell/well) obtained from C57BL/6 mice immunized with the different hybrid mAbs in the presence of CpG ODN or flagellin were resuspended in R10 and incubated with 1μg/mL of either MSP1_19__PADRE or MSP1_19_ recombinant proteins. As negative control, splenocytes were incubated in the absence of any stimulus. Cells were maintained for 96 hours at 37 °C and 5% CO_2_. After the incubation period, culture supernatants were collected, and IL-4, IL-6, IL-17A and IL-10 were detected by CBA (BD Biosciences) exactly as described by the manufacturer. Beads were read in a FACS Canto II flow cytometer (BD biosciences). Results were analyzed using the FCAP Array Software v 2.0 (BD biosciences).

**Sorting of Dendritic Cell Subtypes**

Spleens of 4-6 weeks-old mice were digested in collagenase type IV (Thermo Fisher Scientific) using 100 U/mL during 25 min at 37° C and inactivated with 10 mM EDTA. DCs were enriched from digested cells with a low density gradient of bovine serum albumin (BSA, Amresco). Briefly, cells were resuspented in 30% BSA and cold PBS was carefully overlaid on top. After centrifugation at 2200 rpm during 30 min, the cells in the interface of the low density gradient were collected. Low density gradient cells were incubated with Fc block (BD biosciences) for 40 min on ice and then stained with anti-CD19-Biotin (clone 1D3), anti-CD3-Biotin (clone 145.2C11), anti-CD49b-Biotin (clone DX5), anti-MHCII (I-A/I-E)-FITC (clone M5/114.15.2), anti-CD11c-PE (clone N418), anti-CD8α-PE.Cy7 (clone 53-67) and streptavidin PerCP for 40 min on ice (all antibodies and the streptavidin were purchased from BD Biosciences). After two washes in PBS/BSA 2%/EDTA 2mM CD8α^+^ and CD8α^-^ DCs were incubated with DAPI and sorted in a FACS Aria II (BD Biosciences).

**CD8α^+^ and CD8α^-^ DCs cytokine secretion**

Sorted CD8α^+^ and CD8α^-^ DCs were stimulated *in vitro* with 32 μg/mL CpG ODN 1826 (InvivoGen) or with 1 μg/mL flagellin (produced as described in the materials and methods section) or with medium only for 48 hours at 37 °C and 5% CO_2_. After incubation, cells were centrifuged and the supernatant were freezed at -80°C until the use. TNF-α and IL-6 present on the supernatant were quantified by flex set of BD Cytometric Bead Array (CBA, BD Biosciences) according to the manufacturer's instructions. Acquisition was performed using LSRFortessa (BD Biosciences) and analyses were done in FCAP Array Software V3.0 (BD Bioscience).
